# Students' Knowledge, Attitude, and Practices Regarding Solar Ultraviolet Exposure in Karachi, Pakistan

**DOI:** 10.4314/ejhs.v34i2.3

**Published:** 2024-03

**Authors:** Mubashir Zafar, Tafazzul Hyder Zaidi, Nadira Hyder Zaidi, Muhammad Waqas Nisar Ahmed, Sobia Memon, Faheem Ahmed, Yusra Saleem, Zuhaa Rehman, Anabia Akhlaq, Sana Sajjad Khan, Sana Saleem, Rehan Zaidi

**Affiliations:** 1 Family and Community Medicine Department, College of Medicine, University of Hail, Hail, KSA; 2 Community Medicine Department, Sindh Medical College, Jinnah Sindh Medical University, Karachi, Pakistan; 3 Shaheed Zulfiqar Ali Bhutto Institutes of Science and Technology, Karachi, Pakistan

**Keywords:** ATTITUDE, KNOWLEDGE, PRACTICE, SUNBURN, SUNSCREEN, SUN PROTECTION

## Abstract

**Background:**

Ultraviolet Radiation (UVR) from the sun is a significant environmental component that impacts on human health. Excessive UVR exposure has immediate impacts like burning and tanning, as well as long-term consequences including skin damage, photo-aging, skin malignancies. The objective for this study is to determine the student's knowledge, attitude and practice regarding solar ultraviolet exposure in Karachi, Pakistan.

**Methods:**

A cross-sectional study was done in Public Sector University; total 361 students were recruited through stratified cluster sampling from different colleges of university. Data collection was done by validated structured questionnaire. Logistic regression was used to determine the association of outcome variables with risk factors and p-value of <0.05 as a threshold of statistical analysis.

**Results:**

Most of the students were appropriate knowledge (71%), positive attitude (51%) and safe practice (54%) regarding ultraviolet solar exposure. Almost half (69%) of participants were used sun protective agents. After adjustment of covariate, female gender (OR 2.20 (95% CI 1.29-4.07) (p-value 0.004), significantly associated with in-appropriate knowledge, third year students (OR 2.93 (95% CI 1.01-8.95) p-value 0.048) were significantly associated with negative attitude. Age group 18-21 years (OR 1.75(95% CI 1.43-5.31) p-value 0.003) were significantly associated with un-safe practice regarding solar ultraviolet exposure harmful effects.

**Conclusion:**

This study found that knowledge level was appropriate but attitude and practice level need to improve for prevention of harmful effects of solar ultra violet ray exposure. The study encourages students to be more aware of sun protection behavior in order to avoid the long-term effects of sun exposure.

## Introduction

UVR (ultraviolet radiation) from the sun is a significant environmental component that impacts human health. UVR is divided into three categories according to its energy level: UVA, UVB, and UVC. UVC has the most detrimental effects because of its greater energy level (1). Excessive UVR exposure has immediate impacts like burning and tanning, whereas long-term consequences include skin damage, photo-aging, skin malignancies, immunological suppression, eye damage, and other health problems (2-3). Several studies have published around the world to analyze people's perceptions, attitudes, and practices about sun exposure and related protective strategies. In western countries, various sun protection initiatives have implemented to raise awareness and urge individuals to use sun-protective agents such as sunblock or sunscreen, long-sleeved clothing, wide-brimmed hats, sunglasses(4-5). Study found that those who used sunscreen have a lower risk of cancer compared to than those who do not used sunscreen (6).

A study conducted in developing country found that people were not using sun-protective agents, which indicate a gap in awareness and usage of sunscreen (7). Female student respondents were more likely to be aware of the link between sun exposure and cancer in prior studies. The most well-known benefit reported by the responders while evaluating the level of awareness regarding the effects of the sun among students was the production of Vitamin D (7-8).

Studies have proven inefficient protection with the use of sunscreen under SPF 30 and it is recommended to applying a broad-spectrum sunscreen with SPF 30 or higher (9-11). Sunscreens are classified into organic and inorganic based on their mode of action, inorganic ones, called sunblock, are known for their water-resistant properties (12). The reasons given by participants in various pieces of research for not using sun protection agents include the components such as PABA leading to autoimmune diseases such as Systemic Lupus Erythematosus (13). Other reasons include lack of convenience and lack of knowledge that sunburns can also occur on cloudy days (14).

There are various misconceptions about sun exposure among students and they used different dermatology medications. To best of our knowledge no study was conducted to determine the knowledge and attitude regarding ultraviolet ray exposure among university students. University students especially health related students were primary source of health education regarding solar ultraviolet rays for community of Pakistan. This study helps for making policy to prevent the ultraviolet harmful effects on the skin. Based on the facts and data mentioned above, both internationally and locally, there was a compelling need to encourage people to recognize the harmful consequences of UVR and subsequently take precautionary steps to ensure their safety. For this purpose, our study aimed to assess the knowledge, attitude and practice regarding sun exposure and protection and the detrimental effects of the sun on the skin, as well as their attitudes toward sun-protective measures.

## Methods

**Study setting and sampling technique**: Study was conducted in the public sector university in the different health sciences colleges, total 3000 students were enrolled in these colleges, students were selected through multistage cluster sampling, first identify the health science colleges as a cluster then stratified cluster sampling was used, each academic year classified as strata then simple random sampling was used to select the students from each strata.

**Study design and sample size**: A cross-sectional study was conducted at public sector medical university from April 2022 to August 2022. The study included 361 students from various departments of university.

Sample size calculation was done through Lemeshow Wanga software, 95% confidence interval, 5% margin of error, 38% knowledge level for sun exposure from the previous study (14), total sample was required for this study 361.

**Inclusion and exclusion criteria**: Students were age between 18 years to 25 years, and male and female students were included in the study, any participants were who refused to give informed consent and those students were suffering from solar ultraviolet related diseases were excluded from the study.

**Data collection procedure and tool**: Permission was received from the administration of university and student's leaders for each academic year were trained for data collection, they were responsible to contact the student and give questionnaire with consent form and return back to principal investigator. Students requested to return questionnaire with consent form within a week. The answers for each question were recorded on a separate form for each patient.

**Study tool**: Structured questionnaire was used and this questionnaire was pre tested and it consists of socio-demographic information, knowledge, and attitude and practice section of the questionnaire. Total 8 questions included in the knowledge section, focused on testing the respondent's knowledge regarding UV solar rays, like sources of their information, mode of prevention, sunscreen, risk etc., 8 questions for attitude section which focus on cares of skin, sun protective agents and 9 questions for practice section which focus on the use of sunscreen, time to exposure to sun, etc. in the questionnaire.

**Study variables**: Outcome variable were level of knowledge, attitude and practice. Independent variables were age, gender, academic year of study, past history of skin disease, family history of skin disease.

Each student was interviewed by the investigators who were adequately trained to minimize the investigator bias.

**Scoring**: Knowledge, attitude and practice scales were developed from previous literature (12,14) which contain statement about harmful effects of sun exposure, sunscreen benefit for protection of skin. Attitude scales contain statement behavior regarding sun exposure and practice scales contain statements wearing glass, wearing had and used of sunscreen. Scores were calculated for knowledge, attitude and practice scales. Correct responses were summed on an 8-point rating scale of knowledge, 8-point rating scales of attitude and 9 point rating scale of practice. Those who have a correct answer give one score and incorrect answer gives zero score. Those participants were score 50% correct answer for each knowledge, attitude and practice were given to appropriate, positive attitude and safe practice regarding sun exposure.

**Statistical analysis**: Data entry was done in the Epi data software and analyses were carried out using SPSS software (IBM Corp.), version 26. Data from questionnaire were encoded into database by data encoder. Questionnaire forms and database were checked for completeness daily. Data were entered twice and then cleaned for any missing variables. All the data were supervised by principal investigator on regular basis. Descriptive statistics were obtained i.e. frequencies to determine the percentage of people who have knowledge, attitudes, and practices to protect themselves from harmful UV rays. The study was carried out after obtaining legal permission from the relevant authorities to analyze data and all ethical considerations were observed. The statistical analysis was done with a 95% confidence interval and a p-value of <0.05 was used as the statistical significance threshold.

**Ethical considerations**: The study was approved by the Ethical Review Committee of the Jinnah Sind medical university. (JSMU/IRB/ 2022-604). Before enrolling the study participants, key stakeholders operating in the study areas were informed about the nature and objectives of the research. The participants were informed about the study objective and procedures, and written informed consent was taken from the study participant. The interviews were conducted in a private room for privacy reasons.

## Results

The mean age of our data set was 21.38 years ± 0.487 SD. Out of 361 participants, majority (61.2%) of students were in 18-21 years of age group, 81.4% of the participants were females , 14.4% represents from first year, 21.9% were second year, 16.3% were third year, 39.6% from fourth year and 7.8% from final year of students. Out of all, the majority, 81.2% had no history o f sun disease, and only 9.1% family history of skin disorders (Table 1).

**Table:** Socio-Demographic Characteristics of Students (n=361)

Characteristics	Frequency (%)
**Age (Mean±SD)**	21.38±0.487
18-21	221(61.2)
22-25	140(38.8)
**Gender**	
Female	294(81.4)
Male	67(18.6)
**Academic Year of Study**	
First	52(14.4)
Second	79(21.9)
Third	59(16.3)
Forth	143(39.6)
Fifth	28(7.8)
**History of Skin Disease**	
Yes	68(18.8)
No	293(81.2)
**Family History of Skin Disease**	
Yes	33(9.1)
No	328(90.9)

**Figure 1:**
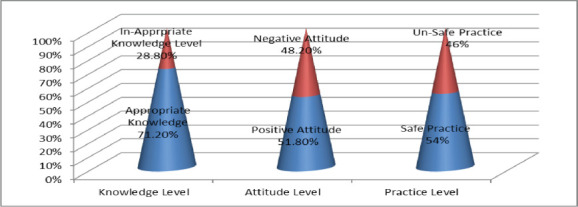
Knowledge, Attitude and Practice Level regarding Sun Exposure among Study Participants

Most (71.2%) of students were appropriate knowledge regarding sun exposure [Figure 1]. When asked about being aware of the harmful effects of UV radiation; 97% said they were aware, When asked sun exposure only you get burn your skin 77.8% were responded yes, When asked if their skin tan; 61.8% said yes. Sun screen application necessary to prevent skin damage, majority (87%) responded yes, long duration sun exposure will cause skin damaged even sunscreen application most (79.5%) of students respond ‘yes’ (Table 2).

**Table 2:** Knowledge, Attitude and Practice regarding Sun Exposure among study participants (n=361)

Question items	n(%)
**Knowledge questions (Correctly Answered)**	
Awareness of Harmful effects of UVR (Ultraviolet Radiation)	350(97)
Prolonged sun exposure is a threat even with the application of high Sun Protection Factor (SPF) agents	287(79.5)
Sun exposure can cause aging, wrinkling, and discoloration of the skin.	323(89.5)
Skin Tan is evidence of skin damage.	223(61.8)
Sun rays have an immunosuppressive effect.	125(34.6)
Light-colored clothing provides better protection against the sun than dark-colored clothing.	286(89.2)
Sunscreen application is necessary to avoid the harmful effects of the sun.	316(87.5)
Sun Exposure only you get burn	281(77.8)
**Attitude questions (Correctly Answered)**	
Are you often worried about protecting your skin from sun exposure? (No)	48(13.3)
Prolonged sun exposure is not good for my body	287(79.5)
Do you think sunscreen harms the skin?	153(42.4)
Sunscreen is not a cosmetic or make-up (Agree)	234(64.8)
Sunscreen is for both men and women (Agree)	137(38)
Sunscreen is like lotion and is not uncomfortable to use (Agree)	127(35.2)
Sunscreen can be worn every day and not only when going to the beach (Agree)	98(27.1)
**Practice questions (Correctly Answered)**	
Wearing Glasses? (No)	237(64.7)
Staying in the shade? (Yes)	249(69)
Reapplying sunscreen every 3-4 hours? (Every day, Sometimes)	259(71.8)
Wearing a wide-brimmed hat (Agree)	231(64)
Avoiding Peak hours of days (Agree)	332(92)
Applying sun protecting agent (Agree)	300(83.7)
Wearing a long-sleeved shirt or short-sleeved shirt with added arm sleeves and long pants (Agree)	350(97)
Do you use any sun-protective agents? (Yes)	249(69)
How often do you use sunscreen? (Every day, Sometimes)	259(71.8)

Half (51%) of students were positive attitude toward sun exposure harmful effects on the skin (Figure 10. When asked about their opinion, if light-colored clothing gives better protection against the sun, 79.2% (n=286) said yes, When asked if sunscreen application is necessary to prevent harmful sun effects, 87.5% were agree this statement (Table 2).

About more than half (54%) of respondents had safe practice for prevention of harmful effects of sun exposure [Figure 1]. When asked if sunscreen should be applied every 3-4 hours, 71.8% were responded yes, 69% of participants were used sun protective agents. Asking about other sun protection practices, about the use of a hat and umbrella, 64% were used it. About using sunglasses, 83.7% said they always used them Full body clothing was always practiced by 97% participants used it (Table 2).

All socio-demographic characteristics were associated with knowledge, attitude and practice regarding sun exposure among study participants. Female gender was more than two times had inappropriate knowledge regarding sun exposure and 78% (OR 0.22 (95% CI 0.11-0.42) p-value 0.000) less likely associated with negative attitude (Table 3).

**Table 3:** Association of Knowledge, Attitude and Practice level with Socio-demographic characteristics among study participants

Socio-Demographic Characteristics	Poor Knowledge Adjusted Odd Ratio (AOR) and Confidence Interval (CI)	p-value	Negative Attitude Adjusted Odd Ratio (AOR) and Confidence Interval (CI)	p-value	Un-Safe Practice Adjusted Odd Ratio (AOR) and Confidence Interval (CI)	p-value
**Age(Years)**						
22-25	1		**1**		**1**	
18-21	1.75(0.93-3.26)	0.079	1.11(0.63-1.98)	0.700	1.75(1.43-5.31)	0.003
**Gender**						
Male	1		1		**1**	
Female	2.29(1.29-4.07)	0.004	1.22(0.11-0.42)	0.080	1.27(0.74-2.18)	0.383
**Academic Year**						
First	1		1		1	
Second	1.68(0.52-5.39)	0.381	2.92(0.93-9.16)	0.065	1.28(0.44-3.73)	0.640
Third	1.99(0.68-5.85)	0.209	2.93(1.01-8.51)	0.048	1.15(0.43-3.11)	0.773
Fourth	1.50(0.51-4.33)	0.454	2.59(0.88-7.58)	0.081	1.04(0.38-2.84)	0.927
Fifth	1.49(0.63-3.56)	0.359	1.80(0.72-4.49)	0.206	1.18(0.51-2.75)	0.689
**History of Skin Disease**						
No	1		1		1	
Yes	0.73(0.40-1.33)	0.310	1.06(0.60-1.87)	0.855	1.01(0.59-1.75)	0.949
**Family History of Skin Disease**						
No	1		1		1	0.653
Yes	2.44(0.92-6.47)	0.072	1.07(0.49-2.31)	0.855	1.18(0.56-2.49)	

## Discussion

This study is one of the few literatures for determining the knowledge, attitude and practice regarding harmful effects of sun exposure in the global warming context. Majority of study participants were appropriate knowledge but attitude and practice were not good regarding harmful effects of sun exposure. The prevalence of sun-protection agent use in students was 69%, which in turn is closely related to the previous study conducted in developing country (15).

In this study knowledge level were appropriate among more than two third of participants. This indicates good awareness regarding harmful effects of sun exposure. In a study conducted in Middle East, students demonstrated excellent knowledge of sun protection practices and identified sun protection as an important modality. The main consequences of sun radiation identified by the students were skin cancer (97.7%), allergies (30.5%), skin aging (72.9%), and blinding (12.9%). Contrarily, in our study, sunburn (60.1%), skin cancer (52.1%), hyperpigmentation (50.7%), tanning (49.3%), eye damage (38.8%), skin aging (35.2%), skin blisters (31.6%), photo-toxicity (23.0%), and vitamin D toxicity (18.3%) were determined to be the harmful effects of sun exposure (4). This to some extent could be justified by the long duration of sun exposure and dry climate of Saudi Arabia in comparison to Karachi, Pakistan.

In this study positive attitude were only fifty percent of study participants. Only 13% of students were not worried about harmful effects of sun exposure. Its means that most of students were practice for sun protection from UV rays. Sunscreen was found to be the most commonly used sun protection modality in our study. When using sunscreen, only 11.9% applied the recommended amount, compared to 29% in previous study of medical students. According to the same study, the reapplication rate after every 3-4 hours was 20%, every 4 hours was 48%, and 17% did not reapply. In our study, 5% reapply every 2 hours, 4.4% every 4 hours, and the majority (54.3%) do not re-apply (11).

In contrast to the knowledge the students have, the practice of sunscreen usage or reapplication is quite low (69%) which is contradict compared to previous study. According to a study conducted among students (16-17), protect against UVR, practice initiated from childhood to young adulthood. Although the majority of our population, 42.7%, were aware of the harmful effects of UV ray exposure although only 8.3% started using sunscreen. This infers the significant gap between having knowledge and the practical implication which could subside if proper measures are taken into consideration. Correlating this result with the other two findings, where the majority, 45.1%, mentioned forgetfulness as the reason for not reapplying (54.3% population do not reapply) sunscreen and social media being the major source of knowing the benefits of sunscreen the gap could be shortened with the proper interventions (18-19). Considering this trend, this study suggested using social media platforms more aptly to spread knowledge among the masses (20-21). One way is to have advertisements that encourage the practice of sun-protective agents. Another way is to have such quotations written in the local language on billboards and bus stops or display graphic posters of dermatological conditions such as skin cancer, skin aging, or sunburns on billboards. Moreover, a study proposed conducting seminars and/or webinars for both, undergraduates and the general public, on the consequences of not using sun protection measures. Also, it should be made known to the public that sunscreen is not the only method of UV protection; they should also use sunglasses, an umbrella, and a hat, as well as prefer full-body clothing when out.

There are several limitations for this study. First this is the cross-sectional study so we do apply the causation rule of temporality. Second, this study had based on self-reported data, therefore, researchers cannot entirely rule out a recall bias or tendency to respond to social desirability. This especially applies to the frequency of sunscreen use during last summer third, this study was conducted during the peak summer season, the results cannot be applied to the participants' entire year of sun protection routine. The study showed an overall good Knowledge, fair awareness and safe Practice level among students concerning the harmful effect of UV rays on the skin. There is a need to clear certain misconceptions to negate the reluctance to use sunscreens by students and in myopic view by the general public. Its also need to health promotion activities to prevent the solar UV ray exposure.
